# Bugs improve nerve regeneration: fasting-induced, microbiome-derived metabolite enhances peripheral nerve regeneration

**DOI:** 10.1038/s41392-022-01186-6

**Published:** 2022-10-05

**Authors:** Zerina Kurtovic, Camilla I. Svensson, Emerson Krock

**Affiliations:** 1 Department. of Physiology and Pharmacology, Centre for Molecular Medicine, Karolinska Institutet, Solna, 171 77 Sweden; 2 Kancera AB, Karolinska Institutet Science Park, Solna, 171 65 Sweden

**Keywords:** Neuroimmunology, Innate immune cells

**Fig. 1:**
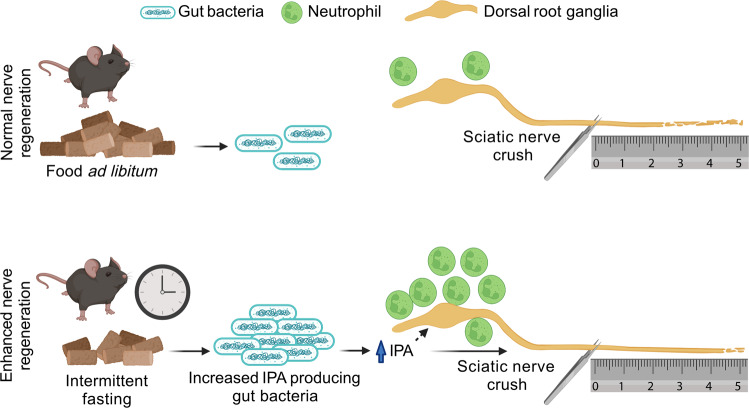
Mice receiving food ad libitum (AL) or as an intermittent fasting (IF) diet were subjected to sciatic nerve crush injury. IF led to enhanced nerve regeneration compared to AL feeding. IF was also associated with elevated serum levels of indole-3-propionic acid (IPA) and IPA-producing gram-positive bacteria in the gut. The IF-induced enhanced nerve regeneration was recapitulated with fecal matter transplants from IF mice to AL mice, gut recolonization with *Clostridium sporogenes*, an IPA-producing bacteria, and systemic IPA delivery. Systemic IPA delivery induced dorsal root ganglia neutrophil infiltration, which was required for IPA enhancement of nerve regeneration. The figure was created with biorender

In a recent study published in *Nature*, Serger et al. connected intermittent fasting (IF) to gut microbiome alterations and enhanced peripheral nerve regeneration following injury.^1^ Fasting has been purported to have neuroregenerative effects, but the underlying mechanisms remained unclear. The authors found that IF-induced elevation of IPA (a microbiome-derived metabolite) promotes neutrophil infiltration into the dorsal root ganglia (DRG), which enhances the regeneration of sciatic nerve fibers (Fig. 1).

Peripheral nerve injuries, acquired through mechanical stress to the nerve, affect more than 20 million people in the US alone, leading to an enormous social and economic burden.^2^ Treatment for nerve injuries involves microsurgery and despite advancements there are still major challenges that prevent a full recovery.^2^ The peripheral nervous system has self-regenerative properties; however, these intrinsic properties are often insufficient and complete recovery is not achieved.^2^ As a consequence, more than 50% of affected individuals are not satisfied with their sensory recovery, have limited motor recovery, and neuropathic pain often develops.^2^ Thus, the need for new therapeutic strategies to promote nerve regeneration is urgent. The regenerative effects of environmental factors like diet and calorie restriction have been previously investigated. In their recent study, Serger et al. make an elegant connection between fasting, the gut microbiome, and neuroimmune pathways, which together improve regeneration in a mouse model of nerve injury.

Serger et al. demonstrate that IF increases axonal regeneration following sciatic nerve crush in rodents. As the first step of discovering the molecular mechanisms that drive enhanced regeneration, the authors compared the serum metabolome of mice fed a regular diet (*ad libitum* (AL)) and mice which had undergone IF, both of which were subjected to sciatic nerve crush. They identified that microbiome-derived, rather than host-derived, metabolites were the most enriched metabolites upon intermittent fasting. Many of the serum enriched metabolites were indole metabolites, which are produced by gram-positive bacteria. The authors then depleted gram-positive bacteria with vancomycin to determine if bacterial-derived metabolites are required for the neuroregenerative effects of IF. Indeed, gram-positive bacteria were necessary as vancomycin abolished the beneficial effects of IF on nerve regeneration. Complimentary, fecal transplants from IF mice to AL mice also promoted post-crush regeneration. Additional metabolomic analyses of serum samples from IF and AL mice treated with vancomycin pointed to indole-3-propionic acid (IPA) as a metabolite relevant for the microbiome-dependent regeneration of nerves. The involvement of the microbiome in nerve generation is also supported by another research group showing that treatment with antibiotics may slow down the nerve regeneration after injury.^3^

Gram-positive bacteria depleted mice that were recolonized with *Clostridium sporogenes*, an IPA-producing bacterial strain, prior to sciatic nerve crush recapitulated IF-enhanced axonal regeneration, but a mutant strain unable to produce IPA did not. Importantly, direct administration of IPA (per oral or intraperitoneally) to mice prior to nerve crush increased nerve regeneration compared to vehicle. In addition to measuring an increased fiber growth, authors injected dextran at the site of injury and IPA-treated mice had more dextran-positive DRG neurons, indicating increased regeneration compared to controls. This, indeed, provides convincing evidence that the microbiome metabolite, IPA, has neuroregenerative effects.

Interestingly, in vitro IPA treatment of DRG neurons did not alter neurite outgrowth, suggesting that neuron-extrinsic mechanisms are driving IF-enhanced axonal regeneration. IPA-independent, unidentified neuron-intrinsic mechanisms could also potentially be contributing to IF-enhance regeneration. Focusing on IPA, the authors investigated non-neuronal mechanisms in the DRG that are modified by IPA following nerve crush. Using RNA-sequencing, they found a transcriptomic signature indicative of neutrophil involvement in IPA-treated mice compared to controls. Immunofluorescent staining revealed increased neutrophil infiltration into the DRG following nerve crush in the IPA-treated animals, further indicating a role for neutrophils. The exact signaling pathway mediating IPA-induced neutrophil infiltration into the DRG has not yet been elucidated. However, neutrophil depletion and blocking neutrophil transmigration into the DRG prevent IPA-induced nerve regeneration, demonstrating that enhanced regeneration requires neutrophil activity. Interestingly, neutrophils were recently found to help resolve pain in humans and mouse models.^4^ Together, these findings suggest that neutrophils have complex neuroprotective effects in the peripheral nervous system, rather than only proinflammatory roles.

Based on the observed interferon-related gene signature in the RNA-seq dataset, the expression of IFNγ was investigated. Immunohistochemical analysis showed that neuronal cell bodies express IFNγ receptor. Importantly, in vitro stimulation of DRG neurons with IFNγ was sufficient to induce neurite outgrowth and this effect was blocked by anti-IFNγ antibodies. Despite macrophages and other immune cells being both IFNγ and IPA responsive, the authors found no evidence of macrophage, T-cell, or NK cell involvement in IF or IPA-enhanced nerve regeneration. It remains possible that there are additional IFNγ regenerative effects via immune cells at non-investigated anatomical sites. In vivo, IFNγ induced expression of the neuronal regeneration markers growth-associated protein-43 and activating transcription factor 3, suggesting IFNγ is a potential link between neutrophils and sensory neurons. Thus, this series of carefully conducted experiments establish a microbiome-dependent immunoneuronal pathway which has potential for therapeutic targeting.

Although IPA-treatment post-injury increased the number of PGP9.5+ fibers that crossed into the epidermis of the skin, there was only a modest reversal of heat hypersensitivity, and no effect was observed on tactile allodynia. It is possible that the repair is limited to certain types of neurons. However, an extensive stratification of the effect of IPA and neutrophil infiltration on different types of fibers remains an open question for future studies. Another unanswered question is whether IF alters peripheral neuronal metabolism to enhance recovery, similar to what has been reported in central nervous system neurons.^5^

This study provides a clear mechanistic link between the neuroprotective effects of intermittent fasting, the gut microbiome, and enhanced peripheral nerve regeneration in mice. These findings further highlight the potential importance of environmental interventions (like diet changes) and microbial products for therapeutic use following nerve injury. Future studies are warranted to identify ways to amplify the neuroregenerative effects of fasting, IPA, and neutrophils and to translate these findings to humans.
